# Noise Reduction in Complex Biological Switches

**DOI:** 10.1038/srep20214

**Published:** 2016-02-08

**Authors:** Luca Cardelli, Attila Csikász-Nagy, Neil Dalchau, Mirco Tribastone, Max Tschaikowski

**Affiliations:** 1 Microsoft Research, 21 Station Road, CB1 2FB, Cambridge, United Kingdom; 2 University of Oxford, Department of Computer Science, Wolfson Building, Parks Road, Oxford OX1 3QD, United Kingdom; 3 Randall Division of Cell and Molecular Biophysics, Institute for Mathematical and Molecular Biomedicine, King’s College London, WC2R 2LS, London, United Kingdom; 4 Fondazione Edmund Mach, Via E. Mach 1, 38010 S. Michele all’Adige (TN), Italy; 5 IMT Institute for Advanced Studies, Piazza S. Francesco 19, 55100 Lucca, Italy

## Abstract

Cells operate in noisy molecular environments via complex regulatory networks. It is possible to understand how molecular counts are related to noise in specific networks, but it is not generally clear how noise relates to network complexity, because different levels of complexity also imply different overall number of molecules. For a fixed function, does increased network complexity reduce noise, beyond the mere increase of overall molecular counts? If so, complexity could provide an advantage counteracting the costs involved in maintaining larger networks. For that purpose, we investigate how noise affects multistable systems, where a small amount of noise could lead to very different outcomes; thus we turn to biochemical switches. Our method for comparing networks of different structure and complexity is to place them in conditions where they produce exactly the same deterministic function. We are then in a good position to compare their noise characteristics relatively to their identical deterministic traces. We show that more complex networks are better at coping with both intrinsic and extrinsic noise. Intrinsic noise tends to decrease with complexity, and extrinsic noise tends to have less impact. Our findings suggest a new role for increased complexity in biological networks, at parity of function.

Cells operate in noisy molecular environments via complex regulatory networks. It is possible to understand how molecular counts are related to noise in specific networks, but it is not generally clear how noise relates to network complexity, because different levels of complexity also imply different overall number of molecules. There is a large literature on how complexity and especially redundancy can increase the robustness of biological systems^1^,^2^,^3^, but it should be emphasized that complexity can also introduce fragility in highly non-linear systems, such as those found in biology^4^,^5^. Other theories claim that complexity beyond a limit can lead to information loss^6^, thus we need a systematic analysis to understand crucial open questions. For a fixed function, does increased network complexity reduce noise, beyond the mere increase of overall molecular counts? If so, complexity could provide an advantage counteracting the costs involved in maintaining larger networks.

For that purpose, we investigate how noise affects multistable systems, where a small amount of noise could lead to very different outcomes; thus we turn to biochemical switches. In a recent paper^7^ two of the authors describe how a classical cell-cycle switch network (*CC*)^8^ approximates the function of a simpler network independently studied in distributed computing: the Approximate Majority algorithm (*AM*)^9^. The theoretical study of *AM* has previously shown that it has some special properties, including asymptotically optimal switching speed and high resistance to noise, which are necessary properties for a “good biological switch”. Although *CC* can approximate the performance of *AM*, there are some differences. We showed that by adding a feedback loop to *CC* that is known to exist in biological networks^10^, we could improve the correspondence between the biological network and *AM*, suggesting that the cell cycle switch can in fact achieve the theoretical *AM*-class performance. Moreover, recent experimental work^11^ has shown that the additional feedback loop (involving the Greatwall kinase) is necessary for the biological function of the cell cycle switch, reinforcing the relationship between biological and computational networks.

A further refinement of the cell cycle switch network has now been proposed^12^, resulting in a highly symmetrical network where all the species are intertwined to regulate each other. We show here that the proposed refinements to the initial cell cycle network actually strengthen the computational connection to the AM algorithm: a *more complex* network becomes *more similar* to a simple network. So similar, in fact, that the correspondence becomes *exact*, and no longer an approximation. Along the way we show also exact correspondence with other networks that have more direct biological significance than *AM*, including various symmetry breaking networks^13^.

## Results

We proceed by first establishing that, for corresponding initial conditions, certain networks of different complexity have compatible function from a deterministic point of view (i.e., by their mass action ODEs, that capture the average behavior of cells). They all realize a fast and robust switching function, and it is possible to make their switching trajectories exactly overlap by a suitable choice of initial conditions and reaction rates. This coincidence of function is based on recent work by some of the authors^14^,^15^ that relates the function of networks of different size based only on structural relationships (that is, based only on reaction graph connectivity and reaction rates).

Since real biological networks have high complexity, but as we have seen, similar dynamical behavior, we may wonder why evolution has selected for such complex systems^16^. To address this question we investigate whether there are stochastic differences in the various networks, and in particular whether the more complex networks gain an advantage in intrinsic noise reduction^17^. We approach this problem by a number of complementary techniques. We compare noise in the various networks by numerically solving the *chemical master equation (CME)*: this gives accurate results for low molecule counts, but becomes quickly unfeasible because the solution depends combinatorially on the number of molecules in the system, and not just on the number of species. We also compare noise in the networks by the *central limit approximation (CLA)*, which becomes more accurate for increasing molecule counts. This technique has the advantage that it can be solved very quickly for large networks and for large initial conditions: the numerical solution is driven by an analytical expression of covariances that depends only on the number of reactions and species (not the number of molecules). The middle ground of intermediate molecule counts (which is of more direct biological relevance) is inaccessible in exact form by either analytical or numerical techniques, but is well bounded by the two above techniques, which give consistent results.

We observe that for equivalent function, more complex networks exhibit a reduction in intrinsic noise. This is not easily attributable simply to the increased total molecule counts^18^,^19^. First, although there are more total molecules in more the complex networks, each species is kept at comparable levels, and secondly, networks of equal total counts exhibit different noise reduction depending on their structure. Overall, the networks of biological origin exhibit better behavior than minimal networks of similar capability.

The manipulation of noise in biological networks is generally of significant interest; for example, it was shown that noise can be beneficial in biological signaling^20^,^21^,^22^,^23^: it can drive a sub-population to behave differently from the majority, so that the population as a whole can better adapt to environmental changes^24^,^25^. We show that intrinsic and extrinsic noise can be adjusted by an increase in network complexity.

### Basic Bistable Networks

We begin by presenting some simple *influence networks*. These are networks of species that catalytically *modify* or *restore* other species; for example, a *modification* could represent an activation of a protein by phosphorylation and a *restoration* could represent a deactivation of the same protein by dephosphorylation^26^. A species in a modified state may have an activity (on other species) that is different from that of the corresponding restored state. It may be that only the restored or modified states are active in such a way, or that both of them are. An influence network does not describe the mechanistic details of the modification/restoration mechanisms, which may vary even within the single biological network that is being modeled. Hiding the mechanisms makes it is easier to see the essential structure of the network^27^, and enables an abstract exploration of relationships between different networks. In order to study the kinetics of these networks, we fix a specific modification/restoration mechanism, so that detailed quantitative comparisons can be made between networks.

We present our influence networks graphically, but our graphical network notation is formal, meaning that each network is an unambiguous depiction of a specific set of chemical reactions. Hence, specific kinetics can be extracted systematically from each network (see S2 Appendix). Well-established approaches to modeling influence networks are similarly formal but are often based on more general classes of kinetic functions that do not directly yield sets of chemical reactions^27^.

**Figure 1:**
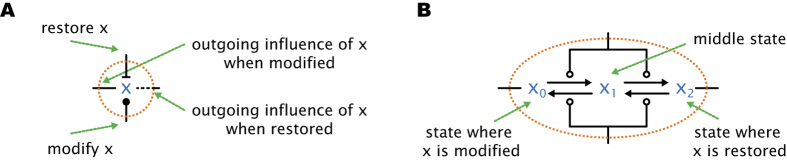
Influence network notation. (**A**) An isolated influence node that would be connected to similar nodes in an influence network. Two of the potential connections represent inputs: modification (ball terminal) and restoration (bar terminal). The other two potential connections represent outputs: activity of the modified state (straight line) and of the restored state (dashed line) on other nodes in the network. (**B**) A specific interpretation of the influence node 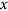 on the left. The node is expanded into a pattern of 3 species and 4 catalytic reactions (the top left hollow ball and arrow, for example, represents the reaction 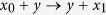 for 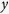 deriving from another node). Species 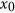 stands for the modified state of 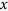, and species 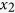 stand for the restored state of 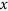. The purpose of species 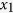 is to act as an internal intermediary in the conversions between 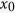 and 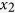, so that the resulting kinetics of the four reactions (in mass action) amounts to a Hill transition between those states. If such a node expansion is applied to all the nodes of an influence network, an unambiguous chemical reaction network results. Remaining degrees of freedom consist in fixing the invariant amounts of 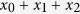 for each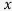, and fixing the rates of the four mass action reactions in each node, at which point the kinetics of the whole network is fixed.

More specifically (Fig. 1), each *influence node* in an influence network represents a species with (at least) two distinct *molecular states*: *modified* and *restored*, which are not themselves considered as separate species. Transitions between these states are interpreted as two-step-modifications; e.g., two sequential phosphorylations or dephosphorylations. Other interpretations are possible (e.g., n-step modification) and should not fundamentally change the kinetics of the network as long as the transitions between the modified and restored molecular states are non-linear: that is, in case of n-step modifications, if n > 1. This specific interpretation of modification/restoration as multi-step modification (which is in line with the cell-cycle model in Ref. 12), based on the concept of multisite phosphorylation^28^, is the basis of our kinetic and stochastic analysis.

Influence nodes are depicted as in Fig. 1A, where a species 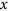 can be modified by species connected to the ball terminal, and restored by species connected to the bar terminal. Modification means that the species 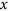 is changed from its restored to its modified molecular state, and conversely for restoration. In turn, 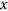 can influence other species, depending on its molecular state, through two kinds of outgoing edges that may connect to other bar or ball terminals. Figure 1B is a view of the same influence node where the molecular states (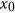 for modified and 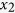 for restored) are explicitly represented; in addition, since we deal with 2-step modification, there is an intermediate molecular state 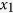 that is not otherwise connected to the rest of the network. The hollow ball terminals here represent simple catalysis. By regulating the rates of flow through 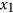 within two orders of magnitude we can obtain a variety of linear, hyperbolic, and sigmoid responses to a linear modification stimulus, such that the response range is equal to the stimulus range (see S1 Appendix).

**Figure 2:**
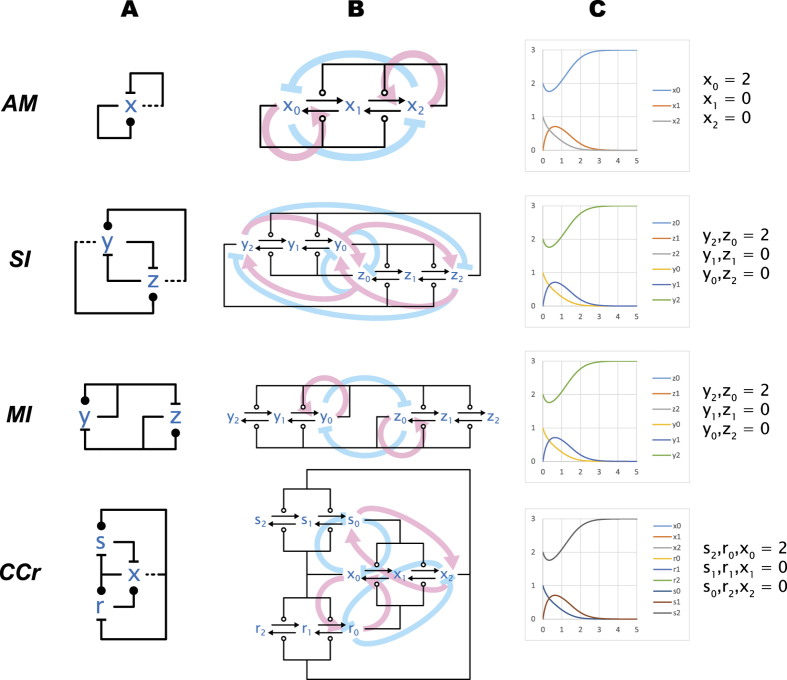
Basic switching networks: deterministic solution. Four networks that are discussed in the text. **(A)** Influence network diagrams. **(B)** Chemical reaction network diagrams and feedback loops. This is the expansion of the influence networks into the corresponding chemical reaction networks according to Fig. 1. Colored arrow illustrate feedback relationships and are not part of the reaction network. **(C)** Numerical solutions of the deterministic kinetics of the networks. First some initial conditions are chosen for AM, and then the initial conditions of the other networks are chosen in such a way that each trace of each of the lower networks retraces exactly one trace of AM. This can be done for any initial conditions chosen for AM, and indicates the potential of each of the lower networks to operate as a simpler switch. Simulation scripts are in S5 Appendix.

Figure 2 shows four networks of interest, where a common characteristic is antagonism between two primary species that results in *mutual inhibition* leading to to bistability: if either species becomes dominant as a population, it becomes self-supporting and forever dominates the other. All of these networks are based on multiple interlocked positive feedback loops. Some of these come in the form of pure positive autocatalytic loops while others are based on double-negative, antagonistic interactions^29^. It has long been known that positive feedback loops are necessary for the emergence of multistability in dynamical systems^30^,^31^,^32^; the interesting feature of these networks is that multiple positive loops are intertwined in a way that generates at least two stable states. The second requirement for multistability is the presence of non-linearity in the system^33^, which comes here in the form of the double-step modifications between modified and restored forms that can be observed in Fig. 2B, where each influence network is expanded into the corresponding chemical reaction networks.

The smallest network, in Fig. 2A(*AM*), has a single influence species; it depicts the Approximate Majority algorithm^9^ and also matches the pattern of the epigenetic switch from Ref. 34. Here the mutual inhibition is internal to 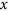, between the 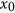 and 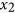 molecular states, which compete for dominance as can be seen in more detail in the corresponding chemical reaction network in Fig. 2B(*AM*).

Figure 2A(*SI*) depicts a network with two influence species, where both modified and restored states are active. The mutual inhibition in SI is achieved by each relevant molecular state of 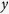 (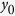, 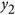) antagonizing the corresponding molecular state of 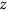 (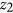, 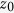), but without direct self-modification. *SI* is inspired by the spatial regulation of the Septation Initiation Network, where two molecules compete for localization at a given cellular structure^13^.

An example of a network where only the modified states have a downstream activity on other species is shown in Fig. 2A(*MI*), where 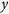 and 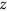 modify themselves and restore the other. This basic arrangement of cross-inhibition of two enzymes in their active forms can be found in many natural and synthetic biological systems, at least in simplified form (in genetic toggle switches, for example, the self-activation loops are usually replaced by inducers or constitutive transcription)^35^,^36^,^37^,^38^,^39^,^40^.

Finally, the network in Fig. 2A(*CCr*) is a modified version of the classical Cell Cycle Switch (*CC*) network^8^. Recognizable are the two smaller feedback loops on the left hand side, the upper one that is double negative, and the lower one that is double positive. In the original *CC* network (not shown), 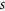 (corresponding to Wee1) and 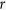 (Cdc25) are directly regulated by x (Cdk), and counterbalanced by fixed biases. Here we connect these counterbalancing reactions to the restored (inactive) form of x to allow for the modification of 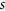 and the restoration of 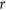 to revert when no stimulus is applied to the system. These additional regulatory feedbacks recapitulate the contributions of known (but more complex) biological feedback loops^41^. As a result, as we shall see, *CCr* can be better related to the other networks than the original *CC*. Moreover, as discussed in Ref. 7, the additional feedbacks result in better switching performance.

### Deterministic Trajectories of Basic Bistable Networks

Each network in Fig. 2 should be understood in a wider context where external signals are applied to it that cause the network to *switch* from one stable state to another (e.g. between 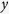-modified and 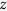-modified in MI). We investigate these external contributions below. Here, however, we begin by studying the core networks, where switching is symbolized by starting from some unstable initial conditions and observing the network settle in one steady state or another.

There is a special relationship between the switching networks in Fig. 2, which we call *emulation*, such that a network *A* can emulate another (typically simpler) network *B*. Emulation means that for *any* choice of rates and initial conditions for *B* we can systematically find rates and initial conditions of *A* such that every trajectory of a species of *A* exactly overlaps a trajectory of a species of *B* (assuming mass action reaction kinetics). That is, when a complex network emulates a simpler network, the complex network behaves redundantly like the simpler network. This relationship is based on a mapping of species of *A* to species of *B* and on other technical requirements^14^, but the important point is that these requirements can be reduced to checks on the stoichiometry, rates, and connectivity of *A* and *B*. That is, they depend only on the structure of *A* and *B*, and do not require examining their kinetic equations.

In Fig. 2C we demonstrate some of these emulation mappings. For sake of example, we fix the initial conditions for *AM* as shown in Fig. 2C(*AM*), and we choose unit rates for all reactions. For each of *MI*, *SI*, and *CCr*, we then map their species into species of *AM* as indicated; for example we map 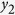 and 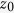 of *MI* to 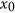 of *AM*, which also determines the initial conditions for 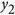 and 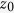 in *MI*. Implicitly, the mappings of species induce mappings of reactions of those networks into reactions of *AM*: these are the natural homomorphic mappings determined by the species mapping. A mapping of species and a mapping of reactions together constitute an emulation, and under these emulation mappings each trajectory of *MI*, *SI*, *CCr* retraces one trajectory of *AM*, as shown in Fig. 2D. Moreover, this exact retracing persists no matter what initial conditions we choose for *AM*, provided we choose initial conditions for the other networks according to the species mapping in Fig. 2C. Similarly, we can vary the reaction rates of *AM* and the retracing persists if we vary the reaction rates of the other networks according to the homomorphic reaction mapping^14^.

The more complex networks will usually have additional behaviors when starting from initial conditions that do not obey the emulation constraints, yielding trajectories that do not match any *AM* trajectories. Hence, emulation is only able to detail a certain *facet* of a complex network. Still, this can be illuminating; for example, we can conclude that although *SI* and *MI* are different networks with different connectivity, found in diverse biological situations, and ultimately exhibiting different kinetics, they still have a common functional facet. Because of the connection of both networks to the *AM* network, that functional facet is the fundamental consensus algorithm that *AM* represents, Approximate Majority^9^, which is an instance of computationally optimal switching. So we immediately know from properties of *AM* that *SI* and *MI* are (at least) bistable and can switch with optimal speed. We can then separately ask whether *SI* and *MI* have other functions in nature, or in what ways they exploit their greater generality, and why biology uses such more complex network instead of a simpler one like AM.

In a similar way, the fact that *CCr* emulates *AM* provides the simplest explanation of the core structure of the G_2_/M cell-cycle switch. That is, it shows that the double-negative and double-positive feedback loops found there^42^, together produce a switching function that is robust and asymptotically optimal: those are properties of *AM* that *CCr* can emulate exactly.

In the following sections we explore the nuances of this commonality, also investigating other networks that fold onto *AM* in the above sense.

### Stochastic Trajectories of Basic Bistable Networks

To reveal possible differences between the networks of Fig. 2 we now study the intrinsic noise characteristics of these networks. A key issue is how to compare stochastic variations in networks that have different numbers of species, and how to choose population sizes for those species so that the comparison is in some sense fair. Our strategy is to compare the networks under an emulation mapping, so that a larger network is reduced in a kinetically compatible way to a smaller network. The emulation mapping constrains the initial conditions for the larger network, as described in the previous section. Since the deterministic trajectories can be interpreted as limits of mean values of stochastic trajectories, we set the stochastic initial condition according to the ones for the deterministic emulation mappings, so that in the limit of large number of molecules the stochastic networks become increasingly similar. Even under those initial conditions, the stochastic trajectories (either their mean or variance) at a particular system size need not coincide.

For example, imagine comparing *AM* with a network that consists just of two separate copies of *AM*. There is a trivial emulation mapping from the two copies to the single *AM* that preserves all deterministic trajectories. In this comparison, the two networks also have the same stochastic behavior for the corresponding species, since the two copies of *AM* are independent. But, more generally, if the two copies of *AM* are intertwined (*SI*, for example, can be seen at two copies of *AM* that have been rewired together), then the stochastic behavior will be affected, and we can compare it fairly with that of *AM*. Moreover, note that *SI* and *MI* have the same number of molecules and reactions, but a different structure, so any differences between them will be attributable to the structure, particularly in conditions where they are both emulating *AM*.

In summary, we compare the means and variances of stochastic networks under emulation mappings. Although the notion of emulation has a deterministic origin, this strategy can be justified by the *fluid limit approximation*, by which the mean of a stochastic process converges to a differential equation^43^. We further analyze the noise by the *central limit approximation*^43^,^44^ (also known as the *linear noise approximation*^45^) by which the variance of a stochastic process can be computed as fluctuations around the deterministic means.

**Figure 3:**
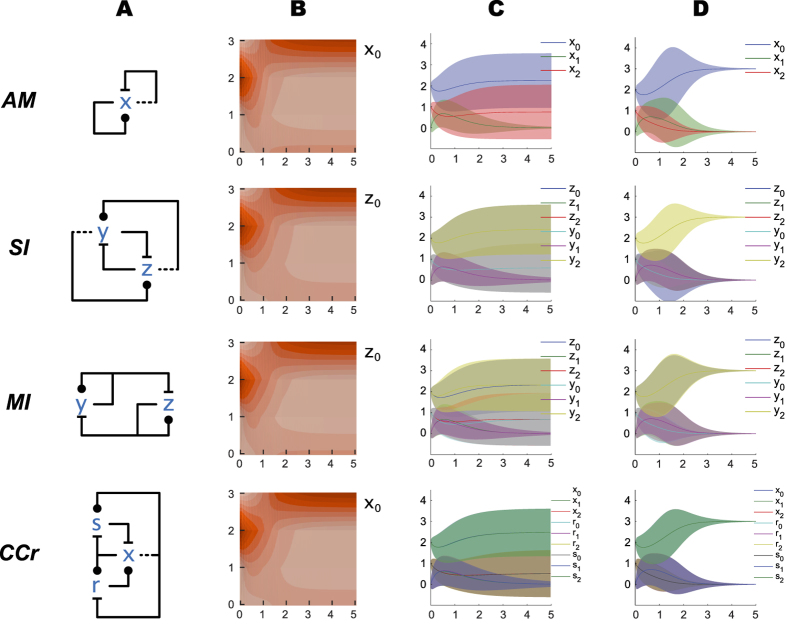
Basic switching networks: stochastic solution. Horizontal axes represent time, vertical axes represent number of molecules. **(A)** Influence networks. **(B)** Chemical Master Equation solution: probability distribution, with color (in 10 bands from light = 0 to dark = 1) indicating the probability that at time t there are y molecules of the single indicated species. **(C)** Chemical Master Equation solution: mean (solid lines) and standard deviation (color bands) for the species in the network. **(D)** Central Limit Approximation solution: mean (solid lines) and standard deviation (color bands) for the species in the network. Simulation scripts are in S5 Appendix.

We begin our analysis by comparing networks via the numerical solution of their Chemical Master Equation (CME), by fixing the emulation mapping and the corresponding initial conditions as in Fig. 2: initial values now represent molecule counts. Figure 3C shows, for example, that solutions of the CME for *AM* and *MI* are very similar in mean and variance at steady state (at time t > 5), with *MI* showing a slight narrowing of variance. Also, *MI* shows some initial divergence of pairs of trajectories due to asymmetries in the first few steps of its stochastic process. *SI* does not exhibit that asymmetry, and at steady state has a markedly narrower variance bands than *AM*; *CCr* has narrower bands still. Note again that *SI* and *MI* have the same number of species and molecules, and that they are compared in conditions where they both emulate *AM*.

Figure 3D shows solutions of the Central Limit Approximation (CLA) for each network; this is a way of approximating the variance in the CME of a finite network by considering a certain limit of increasing system sizes. Unlike the CME, which has one differential equation for each system state, the CLA has one differential equation for each species and one for each pair of species (for their covariance); hence the CLA scales much better than the CME to large networks and initial conditions. We can thus obtain a good approximation of behavior in a range of system sizes that fall beyond what is feasible for the CME, while retaining stochastic information that is lost in the corresponding ODEs.

In some detail, a stochastic process 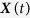 for a CRN associates a discrete random variable over the CRN system states to each time instant 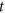. We can consider a sequence 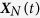 of stochastic processes, with initial states multiplied by an increasing volume 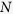, and a normalized sequence 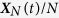 over now continuous random variables. In the limit of 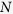 going to infinity, the rescaled mean 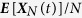 converges to the solution of the ODE system ***v***(*t*) for the original CRN, justifying the approximations 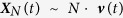. The rescaled variance 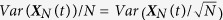, instead converges to the variance of a Gaussian process 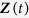, obtained as the limit of the sequence of processes 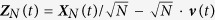. Note that 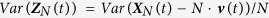, representing the fluctuations of the stochastic process 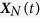 around the deterministic mean 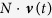. Importantly, 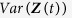
has an analytical form, which enables the fast computation of the CLA. A stochastic process 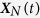 for a given CRN in volume 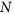 is thus replaced by the stochastic process 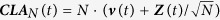, from which we can analytically approximate 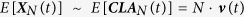 and 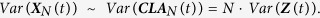 See S3 Appendix for further details.

When comparing the CME (Fig. 3C) to the CLA (Fig. 3D) we should keep in mind two main facts. First, these networks are stochastic and bistable; that is, the distribution of outcomes in final states is highly bimodal at small copy numbers. This can be seen in Fig. 3B, and in the CME solutions of Fig. 3C that have two wide and persisting standard deviation bands for e.g. 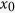 and 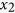 in AM. The bands represent the fact that, with these initial conditions, 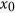 is more likely, but not certain, to settle at the highest level, and 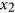 is more likely, but not certain, to settle at the lowest level. In any final state 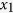 always reaches 0, and this can be seen in its profile: by time 5 is highly likely that a final state has been reached. In a final state there is no noise left in the system (no reactions are enabled), but the distribution of outcomes is bimodal: the standard deviation bands in the final state thus represent uncertainty in outcome rather than sustained noise. A reduction in the widths of these bands means that the outcome is more likely to agree with the initial conditions, that is, with 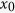 settling at the highest level because it started higher than 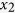. (All the individual stochastic trajectories lie between 0 and 3: bands that go outside of this range are an artifact of plotting the standard deviation around the mean.)

**Figure 4:**
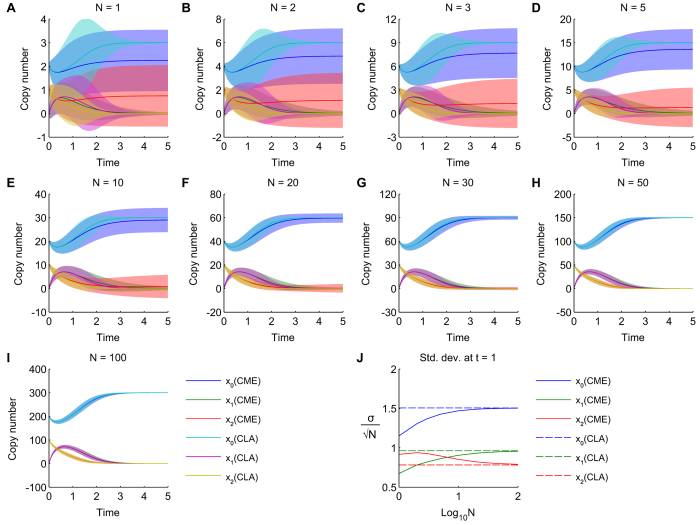
Evolution of CME and CLA solutions with increasing molecular counts. **(A**–**I)** The AM network was analyzed for its stochastic mean (solid lines) and standard deviation (area plots), using the CME and CLA methods. **(J)** Shown are the standard deviations normalized by the square root of the total copy number, N. CME is indicated by solid lines and CLA with dashed lines.

Second, the CLA characterizes a situation at higher copy number, closer to the deterministic limit^45^, where the bimodal distribution becomes gradually more unimodal: at higher copy number 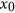 is increasingly more likely to settle at the highest level. This is why in the CLA the standard deviation thins out at greater time points, representing the fact that the distribution becomes unimodal. At earlier time points however 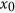 is still noisily finding its way to the top, and it is has non-zero variance. The progression in the CME and CLA from low counts to high counts for the *AM* network can be seen in Fig. 4. The CLA does not provide a good approximation at low molecular counts (since its central limit assumption is violated by bimodal distributions), where we should rely on the CME. The CLA approximation improves at higher counts, and eventually the CME and CLA converge to each other and to the deterministic mean. There is also convergence in the standard deviation about the mean, which become proportional to the square root of the copy number (Fig. 4J).

Careful comparison of the CLA and also of the CME solutions of the various networks of Fig. 3 reveals that the noise is a little bit smaller in the more complex networks. Thus our analysis so far suggests that emulation of a simpler network by a more complex one can perfectly match its deterministic behavior, but the more complex network might reduce the effect of molecular noise. To investigate this hypothesis further we turn to more complex bistable networks.

### Complex cell cycle switch networks

**Figure 5:**
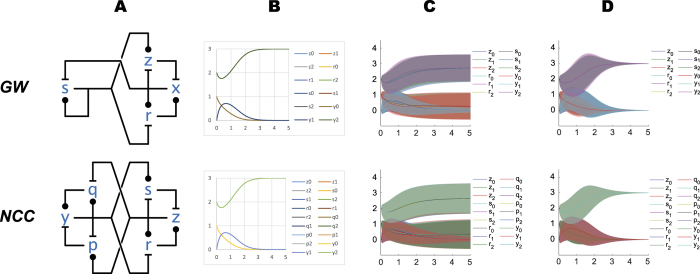
Complex switching networks: stochastic solution. Horizontal axes represent time, vertical axes represent number of molecules. **(A)** Influence networks. **(B)** ODE solutions for comparison with Fig. 2C Chemical Master Equation solution: mean (solid lines) and standard deviation (color bands) for the species in the network. **(D)** Central Limit Approximation solution: mean (black lines) and standard deviation (color bands) for the species in the network. Simulation scripts are in S5 Appendix.

We have shown above that *AM* can summarize the behavior of a minimal cell cycle switch model (*CCr*). Our earlier results show that the original cell cycle network extended with the Greatwall kinase also matches the kinetics of *AM*^7^. That model, *GW*, contains four molecular species (Fig. 5 *GW*), where *x* stands for the core cell cycle regulator kinase Cdk, *z* for its inhibitory kinase Wee1, *r* for the activating phosphatase Cdc25 and *s* for the phosphatase PP2A, which reverts the phosphorylations originally driven by Cdk. The Greatwall kinase (which is not modelled explicit in *GW*) is a component on the pathway from *x* to *s*.

A more recent and detailed model, *NCC* (the Fisher-Krasinska-Coudreuse-Novak Cell Cycle switch^12^), is shown in Fig. 5. The Greatwall kinase is represented by the species *p*, along with the phosphatase PP1 represented by *q*. This network wiring was originally proposed in Ref. 12, but its kinetic behavior was not investigated. Here we show that not only *GW*, but also *NCC* can emulate *AM*, again in the sense that *GW* and *NCC* deterministic traces can reproduce *AM* deterministic traces for all possible combinations of *AM* rates and initial conditions. An example of such traces is shown in Fig. 5B. We further investigate the stochastic behavior of these networks: following the same methods as used above, we calculate the CME and CLA trajectories of these larger systems and compare their standard deviations.

**Figure 6:**
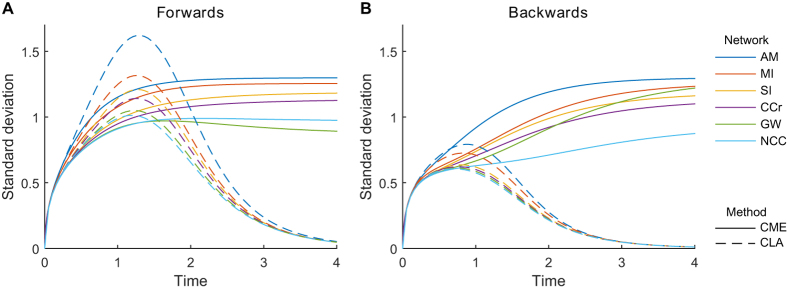
Complexity improves overall performance of the cell cycle switch. The performance of different networks was evaluated by calculating the standard deviation of the main molecular states (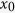 or 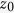, depending on the network) over time. Standard deviations are calculated via numerical integration of the chemical master equation (CME) using the Visual GEC software, and via numerical integration of the central limit approximation (CLA) in Matlab. We investigate switching in one direction or the other by providing different initial conditions that settle (more likely) in different steady states. **(A)** In the forward direction, principal molecular states were initialised at 2 copies, and complementary molecular states were initialised at 1 copy, as shown in Fig. 2C and Fig. 5B. (B) In the reverse direction, principal molecular states were initialised at 1 copy, and complementary molecular states were initialised at 2 copies.

Summarizing, in Fig. 6 we compare the standard deviation of the principal species (those representing Cdk) of all the networks in Figs 3 and 5. Two sets of initial conditions are checked: they each make all the networks exhibit identical deterministic trajectories, as in Figs 2C and 5B. One set of initial condition results in the networks settling with higher probability with the principal species (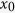 or 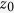) high. The other, symmetrical, set of initial condition results in the networks settling with the principal species low and the complementary species (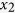 or 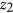) high. Note that the deterministic means of the principal species in the first case match the means of the complementary species in the second case, but this symmetry does not always hold for standard deviation.

The simplest network, *AM*, is the one exhibiting uniformly the highest standard deviation, both in the CME and the CLA. As network complexity increases, there is a trend towards lower standard deviation, with *GW* and *NCC* exhibiting the least amount overall. Note that *GW* has a different relative performance in the two initial conditions. In this kind of comparative analysis, the computation of the CME for networks as complex as *NCC* becomes very expensive and problematic even with very small numbers of molecules of each species: the CLA becomes in practice necessary for a deeper analysis or for larger networks.

So far we have analyzed the behavior of various switching networks by comparing their time evolution, both deterministically and stochastically: this characterizes their switching speed when starting from some arbitrary state. These networks were considered to work in isolation, which is not typical of real biological systems. External stimuli can drive transitions between the two stable states of the cell cycle similarly to many other biological switches^29^. The response of biological switches to external input is traditionally investigated by bifurcation analysis^46^. This technique works only for deterministic systems, thus we need to develop a method for comparing the noise of our models as they are driven back and forth between the two steady states through a hysteresis loop.

**Figure 7:**
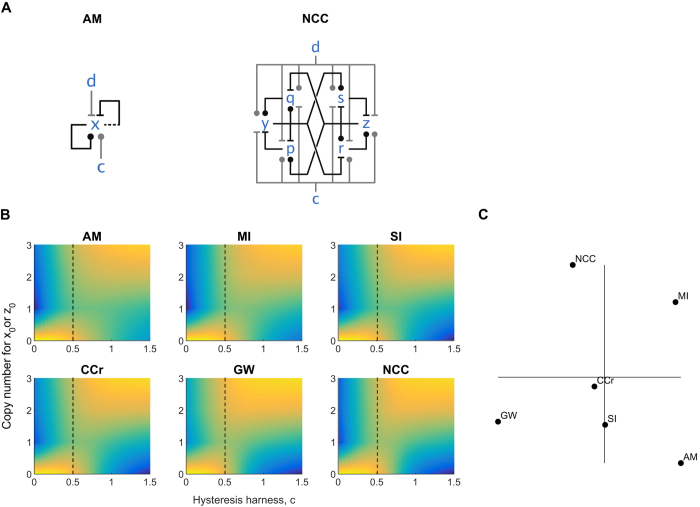
Hysteresis in cell cycle networks. The response to varying input against opposing fixed bias was evaluated as in Ref. 7. (**A)** The networks were placed in hysteresis harnesses, with a stimulus c pushing the switch the one steady-state, and an opposing bias d pushing towards the other steady-state (see Figure S4.1 for additional networks). **(B)** The stationary distribution was computed by integrating the chemical master equation for 100 time units, fixing bias d at 0.5 (indicated by the dashed black line), and varying stimulus c in the interval [0,1.5]. **(C)** The networks were compared by computing a summed Wasserstein metric between each pair of networks. The metric scores were then mapped onto a 2d space using classical multidimensional scaling.

For this kind of analysis, we place the switching networks in hysteresis harnesses (Fig 7A, S4.1) as in Ref. 7, in order to compare their stimulus-response behavior: a varying input stimulus that is pushing the switch in one direction is competing against a fixed bias that is pushing the switch in the opposite direction. Again the question is how to make harnesses for networks of different sizes so that we can compare them in some sense fairly. Our strategy here too is based on emulation: the harnesses must be such that the deterministic equivalence of networks together with their harnesses still holds (see details in S4 Appendix). On that basis we can compare the probability distribution of each network for each amount of stimulus.

The plots of Fig. 7A show the stationary distribution of states at various input levels. The plots look quite similar, but not totally the same. Each network gives a bistable system when the input stimulus is close to the value of the opposite fixed bias (indicated by the dashed lines of Fig. 7B). The width of this bistable region, however, differs between networks. We compare the overall shape of the response by computing the summed Wasserstein metric^47^ (also known as the “earth-mover’s distance”, EMD) between each pair of networks and mapping these into a 2D space (Fig. 7C). This reveals that *AM* is quite different from *GW*, *NCC* and *MI*, but *AM*, *SI*, *CCr* and *NCC* line up on a single line following their increasing complexity.

### Effects of extrinsic noise

Our results so far suggest that the complexity of a network determines how it responds to intrinsic noise caused by low molecule numbers. In real biological systems the external conditions fluctuate as well, resulting in extrinsic noise that could be captured by variations in the rate constants of the models^48^. So far in all our models we have used unity rates for all reactions; next we test how the different networks respond to noise applied to the reaction rates.

**Figure 8:**
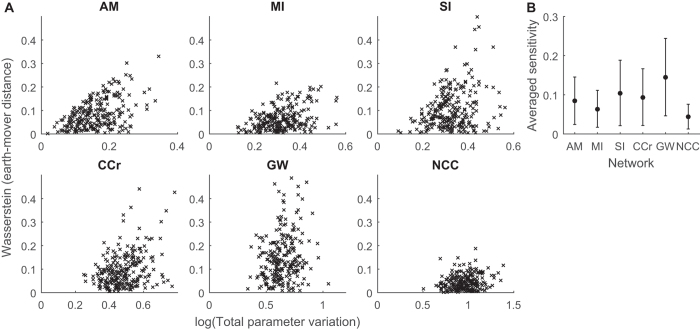
Complexity confers switching networks robustness to extrinsic noise. Extrinsic noise was analyzed by varying the reaction rates of each basal model. **(A)** The extent of 250 reaction rate variations was quantified using the metric in Ref. 49. Variations in network behavior were assessed in comparison to the behavior of the default parameterization, in which all reaction rates are set equal to 1, by simulating the networks under hysteresis harnesses, as in Fig. 7. As before, the variation was quantified using the summed Wasserstein metric (see Appendix S5 for details). **(B)** The network robustness to extrinsic noise is summarized by the mean (circles) and standard deviation (error bars) of the quantifications in **(A)**.

In Fig. 8 we show that, with respect to the basal models (with unit rates), the same level of parameter variation^49^,^50^ leads to smaller deviations in the more complex networks^49^,^50^. To simulate extrinsic noise, the reaction rates are randomly sampled from Gaussian distributions with mean 1 and standard deviation 0.5. The *total* size of the perturbation is then quantified using the metric in Ref. 49, which takes the sum of the deviations on a logarithmic scale. By combining parameter perturbations into a single quantity, networks with different numbers of parameters can be easily compared, though networks with more parameters naturally have a larger total parameter variation. The perturbed system is simulated using hysteresis harnesses (as in Fig. 7A), then compared with the equivalent behavior from the basal rates, again quantified using the Wasserstein metric (see S5 Appendix). This approach resembles classical sensitivity analysis in that we quantify the magnitude of the response to parameter perturbations. However, by generating the perturbations with random variables (taken from a fixed distribution for all parameters), the sensitivity measure is a probability distribution. In Fig. 8B, we use summary statistics to conveniently compare the sensitivity of the networks. Specifically, the mean and standard deviation are computed over all 250 random perturbations. Note that we do not attempt to normalize by the number of reactions/parameters. Interestingly, we see that the six-component *NCC* and, to a lesser extent, the two-component *MI* networks are more robust to extrinsic noise than the one-component *AM*, while other multi-component networks are considerably less robust than *AM* (Fig. 8). Thus, complexity can help to reduce extrinsic noise, but the actual structure of networks is crucial.

## Discussion

We have investigated similarities and differences between robustly switching networks of various complexity. A common feature of all the networks is that they contain at least two positive feedback loops, which are important for their efficient switching dynamics^51^,^52^. We have shown that with corresponding initial conditions all investigated networks show exactly the same average behavior. Moreover, this property extends to different, corresponding, choices of reaction rates. We summarize this kinetic matching between networks by saying that the larger networks emulate (deterministically) the smaller networks^7^.

The fact that *CCr* emulates *AM* provides the simplest explanation of the core structure of the G2/M cell-cycle switch. That is, it shows that the (mysterious) double-negative and double-positive feedback loops found in the control of Cdk activation^53^ produce a switching function that is robust and asymptotically optimal: those are known theoretical properties of *AM*, which *CCr* can emulate exactly. Many other biological switches contain a similar two-positive-feedback loop structure. *AM* has the exact same structure as the epigenetic switching network of nucleosome modification^34^. *SI* resembles the asymmetry regulating switch in fission yeast’s Septation Initiation Network^13^. Many other symmetry breaking systems are controlled by similarly wired networks with various levels of complexity.

Given that baseline of kinetic similarity in mean behavior, we have shown that more complex networks are better in coping with both intrinsic and extrinsic noise. Intrinsic noise tends to decrease with complexity, and extrinsic noise tends to have less impact. To be clear, the absolute noise (the variance, or standard deviation) increases in absolute terms with increasing molecular numbers, as can be seen in Fig. 4 for *AM*, and in fact it is known that noise in protein levels scales with mean protein abundances^54^. At the same time, the relative noise, or more precisely the signal-to-noise ratio, or coefficient of variation, which is given by the ratio of mean to standard deviation, decreases with increasing molecular number. We have compared networks of different complexity were all the species are kept at the same identical levels, and where the more complex networks just have more species (and higher total molecular numbers). We have shown that in those comparable conditions the more complex networks exhibit less absolute intrinsic noise for each species, and less overall variation in response due to extrinsic noise.

It has been argued that in highly optimized biological systems there is a trade-off between robustness (such as robustness to perturbations) and performance, while at the same time there is an evolutionary requirement for both^1^. The cell cycle is surely optimized for biomass production, and presumably operates on the “efficient frontier” of that trade-off^55^. Further, the cell cycle switch implements a computationally optimal algorithm; hence it seems that its performance has not been fundamentally compromised. If performance is held high, then the main way to increase robustness of a system may be to increase the complexity of the biochemical network. This is precisely what we observe in the relationship between the current network of the cell cycle switch and potentially simpler and more ancient versions: performance is maintained, while complexity and robustness is increased. Robustness here can be intended both as resistance to point failures, and resistance to noise.

The real cell cycle switch is likely even more complex than our most detailed model (*NCC*), but must have originated from some simpler network. Larger networks are obviously more expensive to maintain, even just considering the total number of proteins that must be synthesized. Hence, complex networks are less of a burden in situations where resources are not a problem. Based on this we suggest that complex networks are selected to control crucial biological switches in energy rich conditions. In that respect, cell cycle transitions are essential for reproduction and nutrient sensing pathways, where various mechanism ensure that these transitions happen only in good environmental conditions^56^. Thus, the cell cycle switch perfectly matches the conditions that can support complex networks. Conversely, complex networks tend to reduce noise level, and hence for a fixed noise level that can be tolerated, they can support economizing on protein levels.

## Additional Information

**How to cite this article**: Cardelli, L. *et al.* Noise Reduction in Complex Biological Switches. *Sci. Rep.* **6**, 20214; doi: 10.1038/srep20214 (2016).

## Supplementary Material

Supplementary Information
