# Supplementary material for: Noise Reduction in Complex Biological Switches
Source: Sci Rep. 2016 Feb 8;6:20214. doi: 10.1038/srep20214 (PMC4745012; doi:10.1038/srep20214)
Supplement: Supplementary Information [file srep20214-s1.pdf]

Noise Reduction in Complex Biological Switches

Luca Cardelli[1,2,¶,*], Attila Csikász-Nagy[3,4,¶], Neil Dalchau[1,¶], Mirco Tribastone[5,¶], Max Tschaikowski[5,¶]

[1] Microsoft Research Cambridge, United Kingdom; [2] University of Oxford, United Kingdom; [3] King's College London, United Kingdom; [4] Fondazione Edmund Mach, Italy; [5] IMT Institute for Advanced Studies Lucca, Italy; * Corresponding author luca@microsoft.com; ¶ These authors contributed equally to this work.

Supplementary Information

S1 Appendix: The triplet model of influence (adapted from [2] supplement)

An *influence network* is a graph of *influence nodes (species)* and *influence edges (reactions)*. Each influence node x is modeled as three chemical species, denoted x_0, x_1, x_2 (Fig 1). Each influence node can have four terminals: high output (solid line), low output (dashed line), modification input (ball) and restoration input (bar). Influence edges connect two such terminals in one of the four patterns: low-to-modify, low-to-restore, high-to-modify, and high-to-restore, with at most one edge of each kind for each pair of (possibly coincident) nodes.

Each influence node x corresponds to a motif of three chemical species and four chemical reactions (Fig 1). That is, if i is a chemical species connected to the restoration terminal, a is one connected to the modification terminal, and k_{01}, k_{12}, k_{21}, k_{10} are rates associated with the node x, we have the four reactions:

$$x_0 + i \to^{k_{01}} i + x_1, \quad x_1 + i \to^{k_{12}} i + x_2, \quad x_2 + a \to^{k_{21}} a + x_1, \quad x_1 + a \to^{k_{10}} a + x_0$$

This expansion of influence nodes into reaction motifs is sufficient to extract a chemical reaction network from any influence network, taking into account all the influence edges in the network (see S2 Appendix for examples).

We can solve the mass action equations of those four reactions at steady state, with $tot = x_0 + x_1 + x_2$, obtaining x_0 as a function of a and i:

$$x_0 = \frac{k_{10}k_{21}tot\, a^2}{k_{10}k_{21}a^2 + k_{01}k_{21}ai + k_{01}k_{12}i^2}$$

Assuming $i = tot - a$ (restoration decreases as modification increases), we obtain x_0 as a function of a:

$$x_0 = \frac{k_{10}k_{21}tot\, a^2}{(k_{10}k_{21} - k_{01}k_{21} + k_{01}k_{12})a^2 + (k_{01}k_{21} - 2k_{01}k_{12})tot\, a + k_{01}k_{12}tot^2} = \frac{k_1a^2}{k_2a^2 + k_3a + k_4}$$

This is a generalized Hill function of coefficient 2, where the coefficients k_i depend on the four reaction rates and on tot. By regulating the rates of flow through x_1 within two orders of magnitude we can obtain a range of linear, hyperbolic and sigmoid responses in the range $[0..tot]$ to linear modification $a \in [0..tot]$: note that the response range is equal to the stimulus range. Therefore, this motif is sufficiently flexible for the purpose of characterizing intended influence networks.

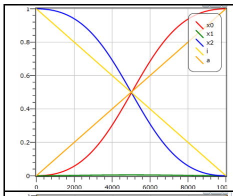	$k_{21} = 0.1$<br>$k_{10} = 10.0$<br>$k_{01} = 0.1$<br>$k_{12} = 10.0$	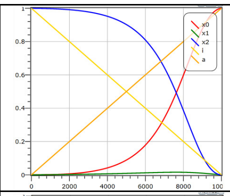	$k_{21} = 0.1$<br>$k_{10} = 1.0$<br>$k_{01} = 0.1$<br>$k_{12} = 10.0$	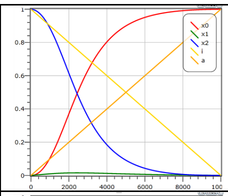	$k_{21} = 0.1$<br>$k_{10} = 10.0$<br>$k_{01} = 0.1$<br>$k_{12} = 1.0$
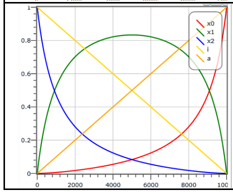	$k_{21} = 10.0$<br>$k_{10} = 1.0$<br>$k_{01} = 10.0$<br>$k_{12} = 1.0$	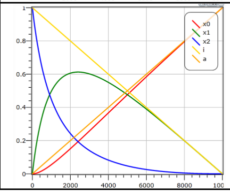	$k_{21} = 10.0$<br>$k_{10} = 1.0$<br>$k_{01} = 1.0$<br>$k_{12} = 1.0$	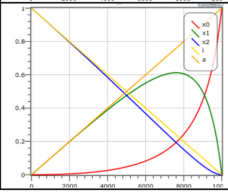	$k_{21} = 1.0$<br>$k_{10} = 1.0$<br>$k_{01} = 10.0$<br>$k_{12} = 1.0$

Figure S1.1. *Steady state transitions from restored to modified with* $tot = 1$ *and* $a \in [0..tot]$.

S2 Appendix: Deterministic analysis

S2.1 Trajectories

For the networks in Fig 2, these systems of ODEs are listed in Fig S2.1, along with conservation equations that result from certain sums of derivatives amounting to zero.

Network	Species	Chemical Reactions (with unit rates)	Dynamical System
AM	$\{x_0, x_1, x_2\}$	$x_2 + x_0 \to x_0 + x_1$<br>$x_1 + x_0 \to x_0 + x_0$<br>$x_0 + x_2 \to x_2 + x_1$<br>$x_1 + x_2 \to x_2 + x_2$	$\dot{x}_0 = x_0x_1 - x_0x_2$<br>$\dot{x}_1 = x_0x_2 + x_2x_0 - x_1x_0 - x_1x_2$<br>$\dot{x}_2 = x_1x_2 - x_2x_0$<br><br>$x_0 + x_1 + x_2 = x$
SI	$\{y_0, y_1, y_2,$<br>$z_0, z_1, z_2\}$	$y_0 + z_0 \to z_0 + y_1$<br>$y_1 + z_0 \to z_0 + y_2$<br>$y_2 + z_2 \to z_2 + y_1$<br>$y_1 + z_2 \to z_2 + y_0$<br><br>$z_2 + y_2 \to y_2 + z_1$<br>$z_1 + y_2 \to y_2 + z_0$<br>$z_0 + y_0 \to y_0 + z_1$<br>$z_1 + y_0 \to y_0 + z_2$	$\dot{z}_0 = z_1y_2 - z_0y_0$<br>$\dot{z}_1 = z_0y_0 + z_2y_2 - z_1y_0 - z_1y_2$<br>$\dot{z}_2 = z_1y_0 - z_2y_2$<br><br>$\dot{y}_0 = y_1z_2 - y_0z_0$<br>$\dot{y}_1 = y_0z_0 + y_2z_2 - y_1z_0 - y_1z_2$<br>$\dot{y}_2 = y_1z_0 - y_2z_2$<br><br>$y_0 + y_1 + y_2 = y$<br>$z_0 + z_1 + z_2 = z$
MI	$\{y_0, y_1, y_2,$<br>$z_0, z_1, z_2\}$	$y_0 + z_0 \to z_0 + y_1$<br>$y_1 + z_0 \to z_0 + y_2$<br>$y_2 + y_0 \to y_0 + y_1$<br>$y_1 + y_0 \to y_0 + y_0$<br><br>$z_2 + z_0 \to z_0 + z_1$<br>$z_1 + z_0 \to z_0 + z_0$<br>$z_0 + y_0 \to y_0 + z_1$<br>$z_1 + y_0 \to y_0 + z_2$	$\dot{z}_0 = z_1z_0 - z_0y_0$<br>$\dot{z}_1 = z_2z_0 + z_0y_0 - z_1z_0 - z_1y_0$<br>$\dot{z}_2 = z_1y_0 - z_2z_0$<br><br>$\dot{y}_0 = y_1y_0 - y_0z_0$<br>$\dot{y}_1 = y_0z_0 + y_2y_0 - y_1z_0 - y_1y_0$<br>$\dot{y}_2 = y_1z_0 - y_2y_0$<br><br>$y_0 + y_1 + y_2 = y$<br>$z_0 + z_1 + z_2 = z$
CCr	$\{x_0, x_1, x_2,$<br>$s_0, s_1, s_2,$<br>$r_0, r_1, r_2\}$	$s_0 + x_0 \to x_0 + s_1$<br>$s_1 + x_0 \to x_0 + s_2$<br>$r_2 + x_0 \to x_0 + r_1$<br>$r_1 + x_0 \to x_0 + r_0$<br><br>$s_2 + x_2 \to x_2 + s_1$<br>$s_1 + x_2 \to x_2 + s_0$<br>$r_0 + x_2 \to x_2 + r_1$<br>$r_1 + x_2 \to x_2 + r_2$<br><br>$x_0 + s_0 \to s_0 + x_1$<br>$x_1 + s_0 \to s_0 + x_2$<br>$x_2 + r_0 \to r_0 + x_1$<br>$x_1 + r_0 \to r_0 + x_0$	$\dot{x}_0 = x_1r_0 - x_0s_0$<br>$\dot{x}_1 = x_0s_0 + x_2r_0 - x_1s_0 - x_1r_0$<br>$\dot{x}_2 = x_1s_0 - x_2r_0$<br><br>$\dot{s}_0 = s_1x_2 - s_0x_0$<br>$\dot{s}_1 = s_0x_0 + s_2x_2 - s_1x_0 - s_1x_2$<br>$\dot{s}_2 = s_1x_0 - s_2x_2$<br><br>$\dot{r}_0 = r_1x_0 - r_0x_2$<br>$\dot{r}_1 = r_2x_0 + r_0x_2 - r_1x_0 - r_1x_2$<br>$\dot{r}_2 = r_1x_2 - r_2x_0$<br><br>$x_0 + x_1 + x_2 = x$<br>$s_0 + s_1 + s_2 = s$<br>$r_0 + r_1 + r_2 = r$

Fig S2.1 *Reaction networks and their differential equations.*

Let $\boldsymbol{v} \in \mathbb{R}^S_{\geq 0}$ be a *state* associating a concentration $\boldsymbol{v}_s \in \mathbb{R}_{\geq 0}$ to each species $s \in S$. Let $f \in \mathbb{R}_{\geq 0} = \mathbb{R}^S_{\geq 0}$ be a *trajectory* of *AM* associating a state $f(t)$ of *AM* to each time point $t \in \mathbb{R}_{\geq 0}$, and $\hat{f} \in \mathbb{R}_{\geq 0} = \mathbb{R}^{\hat{S}}_{\geq 0}$ similarly be a trajectory of *MI*. Suppose that at time 0 we have states $f(0) = \boldsymbol{v}$ for *AM* and $\hat{f}(0) = \hat{\boldsymbol{v}}$ for *MI* such that:

$$\begin{aligned} \boldsymbol{v}_{x_0} &= \hat{\boldsymbol{v}}_{y_2} = \hat{\boldsymbol{v}}_{z_0} \\ \boldsymbol{v}_{x_1} &= \hat{\boldsymbol{v}}_{y_1} = \hat{\boldsymbol{v}}_{z_1} \\ \boldsymbol{v}_{x_2} &= \hat{\boldsymbol{v}}_{y_0} = \hat{\boldsymbol{v}}_{z_2} \end{aligned} \qquad \text{or, informally:} \qquad \begin{aligned} x_0 &= y_2 = z_0 \\ x_1 &= y_1 = z_1 \\ x_2 &= y_0 = z_2 \end{aligned} \qquad \text{(Eq. 1)}$$

So in particular we have $x_0 = z_0$ at time 0. The rate of change of (the concentration $\boldsymbol{v}_{x_0}$of) x_0 at time 0 happens to be the same as the rate of change of z_0, by the assumptions in (Eq. 1):

$$\dot{x}_0 = x_0x_1 - x_0x_2 = z_0z_1 - z_0y_0 = \dot{z}_0$$

Since system evolution is deterministic, we then have that at any future time t, still $x_0 = z_0$ (that is, $f(t)_{x_0} = \hat{f}(t)_{z_0}$). This coincidence of trajectories holds for x_0, z_0, y_2, for x_1, z_1, y_1, and for x_2, z_2, y_0. Therefore, if we start the two networks with initial conditions satisfying (Eq. 1) at time 0, we will have identical time evolution for all the corresponding species of *AM* and *MI*. For example, as in Fig 2, take $x_0 = y_2 = z_0 = 2$, $x_1 = y_1 = z_1 = 0$, $x_2 = y_0 = z_2 = 1$, at time 0. Then the deterministic time evolutions of *AM* and *MI* are identical, with overlapping pairs of traces in *MI*.

The observation that these trajectories coincide is a consequence of a more general phenomenon. Based on a correspondence of species between the networks, such as that in (Eq. 1), all the reaction networks in Fig 2 and Fig 5 can be *folded* (mapped) onto the reaction network of *AM*, in such a way that reactions between corresponding species under the mapping also correspond. This kind of mapping is called a *homomorphism*. But this is not sufficient in general for the coincidence of trajectories. In addition, the mapping must also preserve the stoichiometry of the reactions in a certain way; we call such a mapping a *stoichiomorphism*. Checking that a stoichiomorphism exists between two networks, under a correspondence of species, entails only checking that the stoichiometric constants and reaction rates obey certain constraints: it does not require analyzing the ODEs or any state-dependent property. When a stoichiomorphism exists, we can infer that when starting with coincident initial conditions the networks will evolve along coincident trajectories [2].

More complex networks have more degrees of freedom than *AM*. For example, we can start *MI* in states that do not satisfy (Eq. 1), and therefore we can find traces of *MI* that do not overlap traces of *AM*. But because of the coincidence of trajectories, steady states of *AM* are also steady states of *MI*.

S2.2 Equilibria

By equating the ODEs of each system to 0, we determine the set of equilibria for each network. We wrote a simple algorithm in Mathematica to solve the equations, test the validity of the solution, and then establish linear stability based on the Jacobian matrix of first-order partial derivatives. The results are summarized in the following Table.

Network	Equilibria		Stability
AM	i.	$x_0 = 0, x_1 = N, x_2 = 0$	Unstable
	ii.	$x_0 = 0, x_1 = 0, x_2 = N$	Stable
	iii.	$x_0 = \frac{N}{3}, x_1 = \frac{N}{3}, x_2 = \frac{N}{3}$	Unstable
	iv.	$x_0 = N, x_1 = 0, x_2 = 0$	Stable
SI	i.	$z_0 = 0, z_1 = N, z_2 = 0, y_0 = 0, y_1 = N, y_2 = 0$	Unstable
	ii.	$z_0 = 0, z_1 = 0, z_2 = N, y_0 = N, y_1 = 0, y_2 = 0$	Stable
	iii.	$z_0 = \frac{N}{3}, z_1 = \frac{N}{3}, z_2 = \frac{N}{3}, y_0 = \frac{N}{3}, y_1 = \frac{N}{3}, y_2 = \frac{N}{3}$	Unstable
	iv.	$z_0 = N, z_1 = 0, z_2 = 0, y_0 = 0, y_1 = 0, y_2 = N$	Stable
MI	i.	$z_0 = 0, y_0 = 0$	Unstable
	ii.	$z_0 = N, z_1 = 0, z_2 = 0, y_0 = 0, y_1 = 0, y_2 = N$	Stable
	iii.	$z_0 = 0, z_1 = 0, z_2 = N, y_0 = 0, y_1 = 0, y_2 = N$	Unstable
	iv.	$z_0 = \frac{N}{3}, z_1 = \frac{N}{3}, z_2 = \frac{N}{3}, y_0 = \frac{N}{3}, y_1 = \frac{N}{3}, y_2 = \frac{N}{3}$	Stable
CCr	i.	$x_0 = 0, x_1 = 0, x_2 = 0, s_0 = 0, r_0 = 0$	Unstable
	ii.	$x_0 = N, x_1 = 0, x_2 = 0, s_0 = 0, s_1 = 0, s_2 = N, r_0 = N, r_1 = 0, r_2 = 0$	Stable
	iii.	$x_0 = \frac{N}{3}, x_1 = \frac{N}{3}, x_2 = \frac{N}{3}, s_0 = \frac{N}{3}, s_1 = \frac{N}{3}, s_2 = \frac{N}{3}, r_0 = \frac{N}{3}, r_1 = \frac{N}{3}, r_2 = \frac{N}{3}$	Unstable
	iv.	$x_0 = 0, x_1 = 0, x_2 = N, s_0 = N, s_1 = 0, s_2 = 0, r_0 = 0, r_1 = 0, r_2 = N$	Stable
GW	i.	$z_0 = 0, y_0 = 0, r_0 = 0, s_0 = 0$	Unstable
	ii.	$z_0 = 0, z_1 = 0, z_2 = N, y_0 = 0, r_0 = 0$	Unstable
	iii.	$z_0 = 0, z_1 = 0, z_2 = N, y_0 = N, y_1 = 0, y_2 = 0, r_0 = 0, r_1 = 0, r_2 = N, s_0 = N, s_1 = 0, s_2 = 0$	Stable
	iv.	$z_0 = \frac{N}{3}, z_1 = \frac{N}{3}, z_2 = \frac{N}{3}, y_0 = \frac{N}{3}, y_1 = \frac{N}{3}, y_2 = \frac{N}{3}, r_0 = \frac{N}{3}, r_1 = \frac{N}{3}, r_2 = \frac{N}{3}, s_0 = \frac{N}{3}, s_1 = \frac{N}{3}, s_2 = \frac{N}{3}$	Unstable
	v.	$z_0 = N, z_1 = 0, z_2 = 0, y_0 = 0, y_1 = 0, y_2 = N, r_0 = N, r_1 = 0, r_2 = 0, s_0 = 0, s_1 = 0, s_2 = N$	Stable
NCC	i.	$z_0 = 0, y_0 = 0, r_0 = 0, s_0 = 0, p_0 = 0, q_0 = 0$	Unstable
	ii.	$z_0 = 0, y_0 = 0, y_1 = 0, y_2 = N, r_0 = 0, s_0 = 0, q_0 = 0$	Unstable
	iii.	$z_0 = 0, z_1 = 0, z_2 = N, y_0 = 0, r_0 = 0, p_0 = 0, q_0 = 0$	Unstable
	iv.	$z_0 = 0, z_1 = 0, z_2 = N, y_0 = 0, y_1 = 0, y_2 = N, r_0 = 0, q_0 = 0$	Unstable
	v.	$z_0 = 0, z_1 = 0, z_2 = N, y_0 = N, y_1 = 0, y_2 = 0, r_0 = 0, r_1 = 0, r_2 = N, s_0 = N, s_1 = 0, s_2 = 0, p_0 = 0, p_1 = 0, p_2 = N, q_0 = N, q_1 = 0, q_2 = 0$	Stable
	vi.	$z_0 = \frac{N}{3}, z_1 = \frac{N}{3}, z_2 = \frac{N}{3}, y_0 = \frac{N}{3}, y_1 = \frac{N}{3}, y_2 = \frac{N}{3}, r_0 = \frac{N}{3}, r_1 = \frac{N}{3}, r_2 = \frac{N}{3}, s_0 = \frac{N}{3}, s_1 = \frac{N}{3}, s_2 = \frac{N}{3}, p_0 = \frac{N}{3}, p_1 = \frac{N}{3}, p_2 = \frac{N}{3}, q_0 = \frac{N}{3}, q_1 = \frac{N}{3}, q_2 = \frac{N}{3}$	Unstable
	vii.	$z_0 = N, z_1 = 0, z_2 = 0, y_0 = 0, y_1 = 0, y_2 = N, r_0 = N, r_1 = 0, r_2 = 0, s_0 = 0, s_1 = 0, s_2 = N, p_0 = N, p_1 = 0, p_2 = 0, q_0 = 0, q_1 = 0, q_2 = N$	Stable

Fig S2.2 *Equilibria of reaction networks.*

S3 Appendix: Fluid limit and central limit approximation

In this section we overview the results on fluid limit [Kurtz] and the central limit approximation [5,6] which are used in the paper.

We write a CRN as a Markov population process. This is a continuous-time Markov chain (CTMC) where the state descriptor is given by a vector of non-negative integers that associates with each species its current population of molecules in that state. The state descriptor will be denoted by $\boldsymbol{\xi} = (\xi_s)_{s \in S} \in \Omega$, and the CTMC by $\boldsymbol{X}(t)$, associating a random variable over Ω to each time instant t. Let M be the number of reactions and $N \in \mathbb{N}_{>0}$ refer to the volume of the system. Each reaction is expressed as a jump vector, l_k, with an associated rate function $f_k^N : \Omega^N \to \mathbb{R}$, where $1 \leq k \leq M$. The jump vector records the net stoichiometry of the reaction, whereas the rate function determines the rate at which the reaction happens in each state $\boldsymbol{\xi}$. For instance, let us consider the CRN consisting of reactions $A + B \to^{r_1} C$ and $D \to^{r_2} E$. Then, using the obvious ordering of species, the jump vectors are given by $l_1 = (-1, -1, +1, 0, 0)$ and $l_2 = (0, 0, 0, -1, +1)$ with associated rate functions $f_1^N(\boldsymbol{\xi}) = r_1 \xi_1 \xi_2 / N$ and $f_2^N(\boldsymbol{\xi}) = r_2 \xi_3$, respectively. The idea is to consider a *sequence* of Markov population processes characterized by increasingly larger initial populations of species but such that their initial relative abundances are kept fixed. Let us denote the CTMC sequence by $\{\boldsymbol{X}_N(t), N \in \mathbb{N}_{>0}\}$ (or simply $\boldsymbol{X}_N(t)$). The rate functions have to be in the well-known *density dependent* form, which ensures that the rates of the rescaled CTMC process $\{\boldsymbol{X}_N(t)/N, N \in \mathbb{N}_{>0}\}$ are independent of N. That is, we require that $f_k^N(\boldsymbol{\xi})/N = f_k(\boldsymbol{\xi}/N)$ for all k, N and $\boldsymbol{\xi} \in \Omega^N$, where $f_k : \mathbb{R}^S \to \mathbb{R}$ is a locally Lipschitz function.

In the case of our example, let us fix an *initial concentration* $\boldsymbol{v}(0) \in \mathbb{R}^S_{\geq 0}$ and define the initial population of molecules of the N-th CTMC is by $\lceil N\boldsymbol{v}(0) \rceil$. For instance, let us assume that in our sample CRN we have $\boldsymbol{v}(0) = (0.5, 0.5, 0.0, 0.1, 0.1)$. Then, the first CTMC will have initial condition (1,1,0,1,1) while the 10-th CTMC will have initial condition (5,5,0,1,1). Then, Kurtz's result establishes convergence in probability of the *rescaled* CTMC process $\boldsymbol{X}_N(t)/N$ to $\boldsymbol{v}(t)$ over any finite time time interval, where $\boldsymbol{v}(t)$ is the unique solution of the autonomous ODE system $\dot{\boldsymbol{v}} = F(\boldsymbol{v}) \coloneqq \sum_{k=1}^{M} l_k f_k(\boldsymbol{v})$ subject to initial condition $\boldsymbol{v}(0)$. Generalizing the choice of parameterized rate functions of example to an arbitrary CRN, we observe that the above ODE system corresponds to the usual deterministic mass-action system. This justifies the use of the *fluid approximation* $\mathbb{E}[\boldsymbol{X}_N(t)] \approx N\boldsymbol{v}(t)$ for large values of N (i.e., for large populations of molecules).

Using an analogous setup it is possible to estimate the variance of the population process, with a procedure known as the *central limit* or *linear noise* approximation. For this, starting from $\boldsymbol{X}_N(t)$ we construct a rescaled process centered about the deterministic trajectory. Specifically, we consider the stochastic process $\boldsymbol{Z}_N(t) \coloneqq \left(\zeta_s(t)\right)_{s \in S} \coloneqq \boldsymbol{X}_N(t)/\sqrt{N} - \sqrt{N} \cdot \boldsymbol{v}(t)$. Then, as N goes to infinity this process converges to a Gaussian process $\boldsymbol{Z}(t)$ whose covariance matrix $\boldsymbol{C}(t) \coloneqq Cov(\zeta_s(t), \zeta_{s'}(t))_{s,s' \in S}$ satisfies the ODE system (in matrix notation)

$$\frac{d\boldsymbol{C}(t)}{dt} = \boldsymbol{J_F}\big(\boldsymbol{v}(t)\big)\boldsymbol{C}(t) + \boldsymbol{C}(t)\boldsymbol{J_F^T}\big(\boldsymbol{v}(t)\big) + \boldsymbol{G}(\boldsymbol{v}(t))$$

with initial condition $\boldsymbol{C}(0) = 0$, where $\boldsymbol{J_F}\big(\boldsymbol{v}(t)\big)$ is the Jacobian of the ODE vector field F evaluated at the solution of the fluid limit $\boldsymbol{v}(t)$ and $\boldsymbol{G}(\boldsymbol{x}) \coloneqq \sum_{\boldsymbol{k=1}}^{\boldsymbol{M}} l_k l_k^T f_k(\boldsymbol{x})$. This justifies the use of the approximation for the variance $\mathbb{V}\big[\boldsymbol{X}_N(t)/\sqrt{N} - \sqrt{N} \cdot \boldsymbol{v}(t)\big] \approx \mathbb{V}[\boldsymbol{Z}(t)]$ for large N.

Plots of the standard deviation bands around the ODE solution for selected influence networks are shown below.

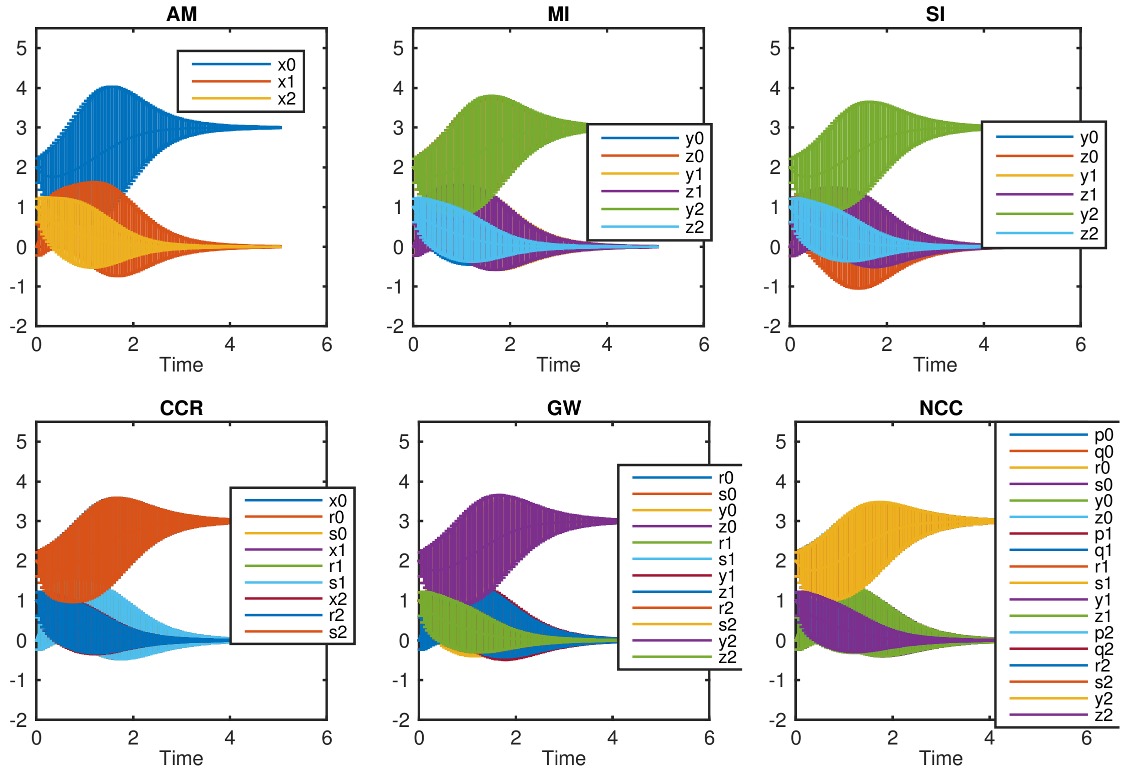

Fig S3.1. *Standard deviation band about the ODE solution obtained by central limit approximation.*

S4 Appendix: Transitions between steady states

We place networks in hysteresis harnesses (Fig S4.1) and we verify that they still behave correspondingly (Fig S4.2). This is in support of the discussion in Section “Complex cell cycle switch networks”.

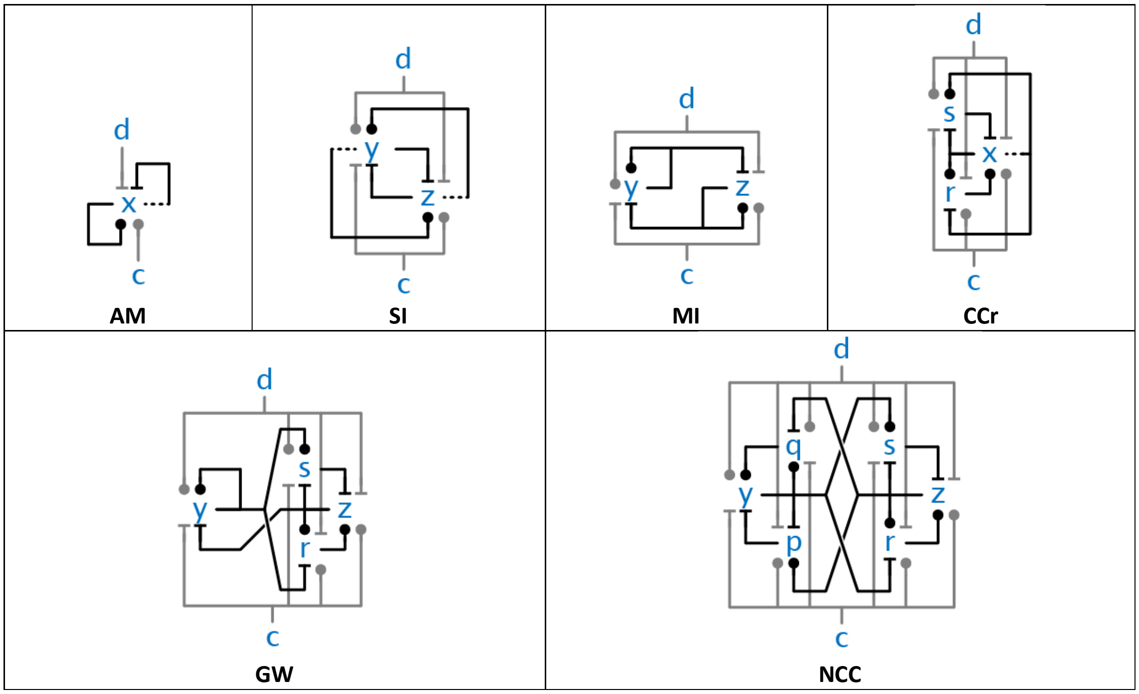

Fig S4.1 – Harnesses for stimulus-response study. *The AM network is augmented with a fixed bias d held at an intermediate level, and an opposite varying stimulus c. In absence of the stimulus c, the fixed bias d pushes the switch in one steady state. As the level of c increases it overcomes d and flips the switch in the other steady state. As c then decreases, d overcomes c to switch back, but at a different switching point, exhibiting hysteresis as seen in Fig 7. The other networks, which all emulate AM, are placed in harnesses such that the network with its harness emulates AM with its harness.*

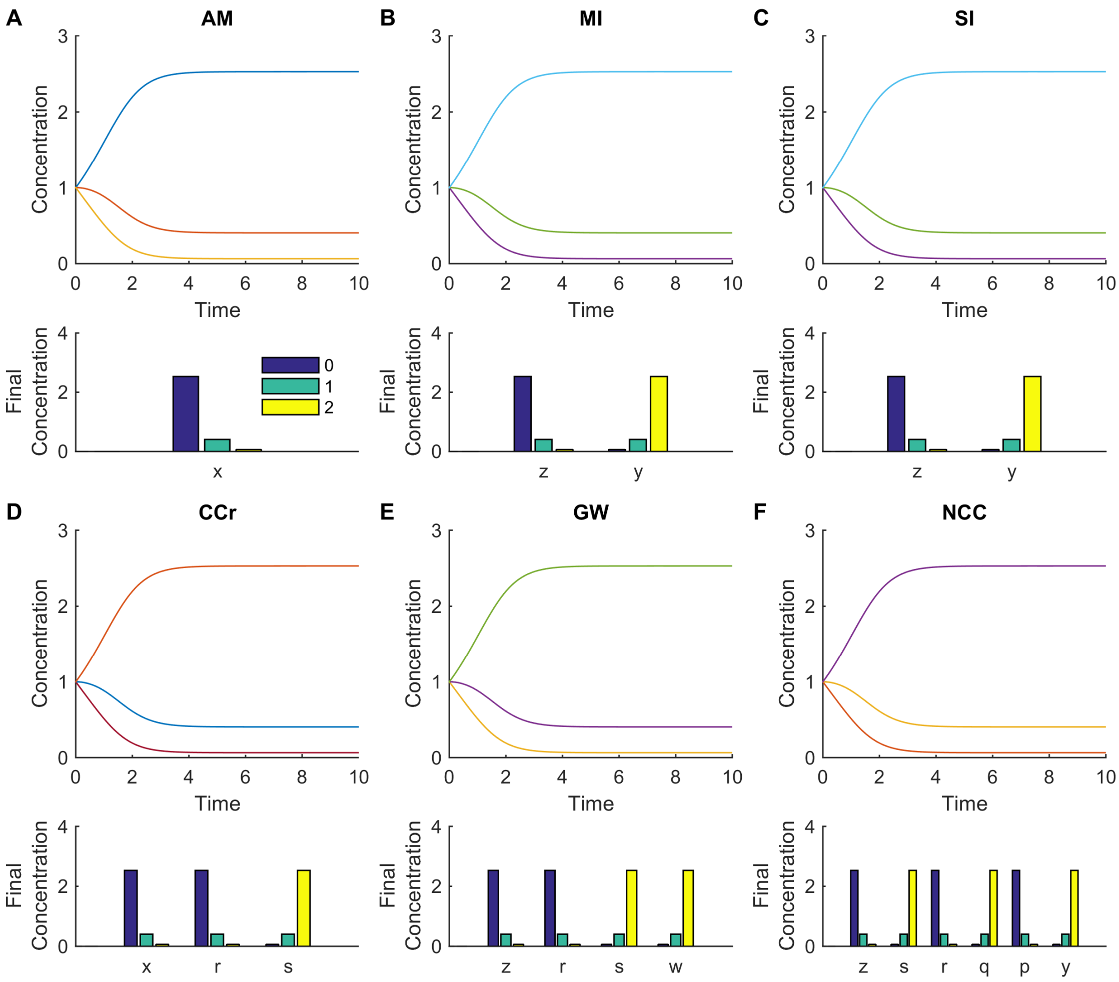

Fig S4.2 – *Emulation of external inputs to the system. To ensure that the hysteresis harnesses applied to each network were equivalent, ODE simulations were performed for each network (**A**: AM, **B**: MI, **C**: SI, **D**: CCr, **E**: GW, **F**: NCC), with the input stimulus set to 1 and the backwards fixed bias set to 0.5. For each network, the dynamics are shown for each species over time (top panels), and form three groups, due to emulation. To visualize which species belong to each group, the final concentrations for each influence node are compared with a bar chart (bottom panels): the bar triplets correspond to the triplets of variables for each influence species.*

S5 Appendix: Comparing stationary distributions with the Wasserstein metric

The Wasserstein metric (also known as the Earth Mover's Distance, EMD) can be used to quantify the distance between two distributions. Informally, it can be interpreted as the cost of converting one pile of earth into another, hence the EMD moniker. It considers both the amount of earth that must be moved and how far it must be moved. For discrete distributions over a single variable, it can be computed efficiently with a simple recursive algorithm (https://en.wikipedia.org/wiki/Earth_mover%27s_distance).

Given two discrete domain distributions $u, v \in \mathbb{R}_{+}^{n}$, the EMD can be computed as:

$$EMD_0 = 0$$

$$EMD_{i+1} = u_i + EMD_i - v_i$$

$$EMD = \sum_{i}^{n} |EMD_i|$$

We apply this distance measure to pairs of hysteresis plots from Figure 7A by computing the distance between the stationary distributions at a given stimulus (once per value of *c*), then taking the sum. Similarly, this summed EMD is used for analyzing extrinsic noise in Figure 8A, by comparing the stationary distributions in the hysteresis plots between basal parameter values (all equal to 1) and randomly perturbed values.

S6 Appendix: Numerical simulation scripts

<table>
<tr><th></th><th>A Influence Network Diagram</th><th>B LBS ODE simulation</th><th>C LBS CME simulation</th></tr>
<tr><td>AM</td><td>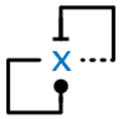
</td><td>(* AM *)
directive sample 5.0 100
directive simulation deterministic

rate k = 1;

init x0 2 |
init x1 0 |
init x2 1 |

x0 + x2 ->{k} x2 + x1 |
x2 + x0 ->{k} x0 + x1 |
x1 + x0 ->{k} x0 + x0 |
x1 + x2 ->{k} x2 + x2</td><td>(* AM cme *)
directive sample 5.0 100
directive simulation cme

rate k = 1;

init x0 2 |
init x1 0 |
init x2 1 |

x0 + x2 ->{k} x2 + x1 |
x2 + x0 ->{k} x0 + x1 |
x1 + x0 ->{k} x0 + x0 |
x1 + x2 ->{k} x2 + x2</td></tr>
<tr><td>SI</td><td>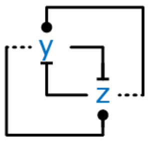
</td><td>(* SI *)
directive sample 5.0 100
directive simulation deterministic

rate k = 1;

init z0 2 |
init z1 0 |
init z2 1 |
init y0 1 |
init y1 0 |
init y2 2 |

z0 + y0 ->{k} y0 + z1 |
z1 + y0 ->{k} y0 + z2 |
z2 + y2 ->{k} y2 + z1 |
z1 + y2 ->{k} y2 + z0 |

y0 + z0 ->{k} z0 + y1 |
y1 + z0 ->{k} z0 + y2 |
y2 + z2 ->{k} z2 + y1 |
y1 + z2 ->{k} z2 + y0</td><td>(* SI cme *)
directive sample 5.0 100
directive simulation cme

rate k = 1;

init z0 2 |
init z1 0 |
init z2 1 |
init y0 1 |
init y1 0 |
init y2 2 |

z0 + y0 ->{k} y0 + z1 |
z1 + y0 ->{k} y0 + z2 |
z2 + y2 ->{k} y2 + z1 |
z1 + y2 ->{k} y2 + z0 |

y0 + z0 ->{k} z0 + y1 |
y1 + z0 ->{k} z0 + y2 |
y2 + z2 ->{k} z2 + y1 |
y1 + z2 ->{k} z2 + y0</td></tr>
<tr><td>MI</td><td>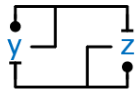
</td><td>(* MI *)
directive sample 5.0 100
directive simulation deterministic

rate k = 1;

init z0 2 |
init z1 0 |
init z2 1 |
init y0 1 |
init y1 0 |
init y2 2 |

z2 + z0 ->{k} z0 + z1 |
z1 + z0 ->{k} z0 + z0 |
y0 + z0 ->{k} z0 + y1 |
y1 + z0 ->{k} z0 + y2 |

y2 + y0 ->{k} y0 + y1 |
y1 + y0 ->{k} y0 + y0 |
z0 + y0 ->{k} y0 + z1 |
z1 + y0 ->{k} y0 + z2</td><td>(* MI cme *)
directive sample 5.0 100
directive simulation cme

rate k = 1;

init z0 2 |
init z1 0 |
init z2 1 |
init y0 1 |
init y1 0 |
init y2 2 |

z2 + z0 ->{k} z0 + z1 |
z1 + z0 ->{k} z0 + z0 |
y0 + z0 ->{k} z0 + y1 |
y1 + z0 ->{k} z0 + y2 |

y2 + y0 ->{k} y0 + y1 |
y1 + y0 ->{k} y0 + y0 |
z0 + y0 ->{k} y0 + z1 |
z1 + y0 ->{k} y0 + z2</td></tr>
<tr><td>CCr</td><td>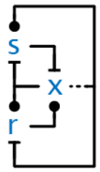
</td><td>(* CCr *)
directive sample 5.0 100
directive simulation deterministic

rate k = 1;

init x0 2 |
init x1 0 |
init x2 1 |
init r0 2 |
init r1 0 |
init r2 1 |
init s0 1 |
init s1 0 |
init s2 2 |

r2 + x0 ->{k} x0 + r1 |
r1 + x0 ->{k} x0 + r0 |
s0 + x0 ->{k} x0 + s1 |
s1 + x0 ->{k} x0 + s2 |

s2 + x2 ->{k} x2 + s1 |
s1 + x2 ->{k} x2 + s0 |
r0 + x2 ->{k} x2 + r1 |
r1 + x2 ->{k} x2 + r2 |

x2 + r0->{k} r0 + x1 |
x1 + r0->{k} r0 + x0 |
x0 + s0->{k} s0 + x1 |
x1 + s0->{k} s0 + x2</td><td>(* CCr cme *)
directive sample 5.0 100
directive simulation cme

rate k = 1;

init x0 2 |
init x1 0 |
init x2 1 |
init r0 2 |
init r1 0 |
init r2 1 |
init s0 1 |
init s1 0 |
init s2 2 |

r2 + x0 ->{k} x0 + r1 |
r1 + x0 ->{k} x0 + r0 |
s0 + x0 ->{k} x0 + s1 |
s1 + x0 ->{k} x0 + s2 |

s2 + x2 ->{k} x2 + s1 |
s1 + x2 ->{k} x2 + s0 |
r0 + x2 ->{k} x2 + r1 |
r1 + x2 ->{k} x2 + r2 |

x2 + r0->{k} r0 + x1 |
x1 + r0->{k} r0 + x0 |
x0 + s0->{k} s0 + x1 |
x1 + s0->{k} s0 + x2</td></tr>
</table>

<table>
<tr>
<td>GW</td>
<td>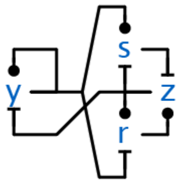
</td>
<td>(* GW *)
directive sample 5.0 100
directive simulation deterministic

rate k = 1;

init z0 2 |
init z1 0 |
init z2 1 |
init r0 2 |
init r1 0 |
init r2 1 |
init s0 1 |
init s1 0 |
init s2 2 |
init y0 1 |
init y1 0 |
init y2 2 |

y0 + z0 ->{k} z0 + y1 |
y1 + z0 ->{k} z0 + y2 |
r2 + z0 ->{k} z0 + r1 |
r1 + z0 ->{k} z0 + r0 |
s0 + z0 ->{k} z0 + s1 |
s1 + z0 ->{k} z0 + s2 |

z2 + r0 ->{k} r0 + z1 |
z1 + r0 ->{k} r0 + z0 |

z0 + s0 ->{k} s0 + z1 |
z1 + s0 ->{k} s0 + z2 |

y2 + y0 ->{k} y0 + y1 |
y1 + y0 ->{k} y0 + y0 |
r0 + y0 ->{k} y0 + r1 |
r1 + y0 ->{k} y0 + r2 |
s2 + y0 ->{k} y0 + s1 |
s1 + y0 ->{k} y0 + s0</td>
<td>(* GW cme *)
directive sample 5.0 100
directive simulation cme

rate k = 1;

init z0 2 |
init z1 0 |
init z2 1 |
init r0 2 |
init r1 0 |
init r2 1 |
init s0 1 |
init s1 0 |
init s2 2 |
init y0 1 |
init y1 0 |
init y2 2 |

y0 + z0 ->{k} z0 + y1 |
y1 + z0 ->{k} z0 + y2 |
r2 + z0 ->{k} z0 + r1 |
r1 + z0 ->{k} z0 + r0 |
s0 + z0 ->{k} z0 + s1 |
s1 + z0 ->{k} z0 + s2 |

z2 + r0 ->{k} r0 + z1 |
z1 + r0 ->{k} r0 + z0 |

z0 + s0 ->{k} s0 + z1 |
z1 + s0 ->{k} s0 + z2 |

y2 + y0 ->{k} y0 + y1 |
y1 + y0 ->{k} y0 + y0 |
r0 + y0 ->{k} y0 + r1 |
r1 + y0 ->{k} y0 + r2 |
s2 + y0 ->{k} y0 + s1 |
s1 + y0 ->{k} y0 + s0</td>
</tr>
<tr>
<td>NCC</td>
<td>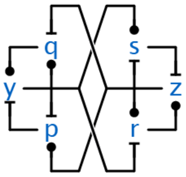
</td>
<td>(* NCC *)
directive sample 5.0 100
directive simulation deterministic

rate k = 1;

init z0 2 |
init z1 0 |
init z2 1 |

init s0 1 |
init s1 0 |
init s2 2 |

init r0 2 |
init r1 0 |
init r2 1 |

init q0 1 |
init q1 0 |
init q2 2 |

init p0 2 |
init p1 0 |
init p2 1 |

init y0 1 |
init y1 0 |
init y2 2 |

z0 + s0 ->{k} s0 + z1 |
z1 + s0 ->{k} s0 + z2 |
z2 + r0 ->{k} r0 + z1 |
z1 + r0 ->{k} r0 + z0 |

s2 + y0 ->{k} y0 + s1 |
s1 + y0 ->{k} y0 + s0 |
s0 + z0 ->{k} z0 + s1 |
s1 + z0 ->{k} z0 + s2 |

r2 + z0 ->{k} z0 + r1 |
r1 + z0 ->{k} z0 + r0 |
r0 + y0 ->{k} y0 + r1 |
r1 + y0 ->{k} y0 + r2 |

y2 + q0 ->{k} q0 + y1 |
y1 + q0 ->{k} q0 + y0 |
y0 + p0 ->{k} p0 + y1 |
y1 + p0 ->{k} p0 + y2 |

q2 + y0 ->{k} y0 + q1 |
q1 + y0 ->{k} y0 + q0 |
q0 + z0 ->{k} z0 + q1 |
q1 + z0 ->{k} z0 + q2 |

p2 + z0 ->{k} z0 + p1 |
p1 + z0 ->{k} z0 + p0 |
p0 + y0 ->{k} y0 + p1 |
p1 + y0 ->{k} y0 + p2</td>
<td>(* NCC cme *)
directive sample 5.0 100
directive simulation cme

rate k = 1;

init z0 2 |
init z1 0 |
init z2 1 |

init s0 1 |
init s1 0 |
init s2 2 |

init r0 2 |
init r1 0 |
init r2 1 |

init q0 1 |
init q1 0 |
init q2 2 |

init p0 2 |
init p1 0 |
init p2 1 |

init y0 1 |
init y1 0 |
init y2 2 |

z0 + s0 ->{k} s0 + z1 |
z1 + s0 ->{k} s0 + z2 |
z2 + r0 ->{k} r0 + z1 |
z1 + r0 ->{k} r0 + z0 |

s2 + y0 ->{k} y0 + s1 |
s1 + y0 ->{k} y0 + s0 |
s0 + z0 ->{k} z0 + s1 |
s1 + z0 ->{k} z0 + s2 |

r2 + z0 ->{k} z0 + r1 |
r1 + z0 ->{k} z0 + r0 |
r0 + y0 ->{k} y0 + r1 |
r1 + y0 ->{k} y0 + r2 |

y2 + q0 ->{k} q0 + y1 |
y1 + q0 ->{k} q0 + y0 |
y0 + p0 ->{k} p0 + y1 |
y1 + p0 ->{k} p0 + y2 |

q2 + y0 ->{k} y0 + q1 |
q1 + y0 ->{k} y0 + q0 |
q0 + z0 ->{k} z0 + q1 |
q1 + z0 ->{k} z0 + q2 |

p2 + z0 ->{k} z0 + p1 |
p1 + z0 ->{k} z0 + p0 |
p0 + y0 ->{k} y0 + p1 |
p1 + y0 ->{k} y0 + p2</td>
</tr>
</table>

Fig S5.1 – Networks and their simulation scripts. A *Influence network diagram.* ***B*** *LBS script for ODE simulation.* ***C*** *LBS script for CME simulation.*

LBS is the Language for Biochemical Systems:
http://homepages.inf.ed.ac.uk/gdp/publications/Lang_Bio_Sys_Design_Spec.pdf,
which is part of the GEC simulation tool: http://research.microsoft.com/gec.
